# Marion Eugene Martin

**Published:** 1918-10

**Authors:** 


### OBITUARY

Readers are requested to report promptly the death of all physicians in Western New York, or former residents of this region, or graduates of any medical school in Western New York, and to notify the families of the deceased of our desire to publish adequate Obituary notices.

**Marion Eugene Martin,** Attica, N. Y.; Buffalo, N. Y., University, 1892; aged 54; a Fellow of the American Medical Association; for several years coroner of Wyoming County; died at his home, August 14, from heart disease.

